# Argentinian Spanish Adaptation of the Activities-specific Balance Confidence (ABC) Scale

**DOI:** 10.7759/cureus.85334

**Published:** 2025-06-04

**Authors:** Daniel H Verdecchia, Agustina M Monzon, Tatiana Dias de Carvalho, Ladislao P Diaz Ballve, Diego A Felici

**Affiliations:** 1 Department of Health Sciences, Universidad Nacional de La Matanza, San Justo, ARG

**Keywords:** accidental falls, aged, patient health questionnaire, postural balance, trust

## Abstract

Background

There are diverse factors that increase the risk of falls for older adults. One of the most important is a lack of confidence when performing everyday activities. The Activities-specific Balance Confidence (ABC) Scale, developed in Canada, is used to quantify the perception of confidence in balance on the part of an older adult when carrying out habitual activities. As yet, an Argentine version of the ABC does not exist. The objective of this study was to translate, culturally adapt, and evaluate the internal consistency of the ABC Scale questionnaire for use in the Argentine adult population over 65 years of age.

Materials and methods

The translation and transcultural adaptation process followed the World Health Organization guidelines for instrument adaptation. This included (1) independent forward translation by bilingual experts; (2) synthesis by a multidisciplinary panel; (3) back-translation to verify conceptual equivalence; and (4) pretesting with cognitive debriefing in the target population. Participants were community-dwelling adults aged 65 years or older who were literate and capable of walking independently or with assistive devices. Internal consistency was evaluated using Cronbach’s alpha coefficient.

Results

A total of 30 subjects with a median age of 69.5 years participated; 70% of these were women. Five of the 16 items in the ABC were modified for the transcultural adaptation. The median of the ABC was 83.6% (IQR: 67.2-98.4). A roof effect was observed in five (16.7%) of the subjects. The minimum value was 31.2 points. The result for internal consistency was acceptable (Cronbach’s alpha: 0.92).

Conclusions

The original version of the ABC Scale was successfully translated and culturally adapted for use in the older adult population in Argentina. The Argentine version demonstrated excellent internal consistency and was easily understood and completed by participants, confirming its feasibility in clinical settings. This culturally adapted tool provides healthcare professionals with a reliable instrument to assess balance confidence in older adults. Further studies are needed to validate its use in clinical populations with specific health conditions and to explore its applicability in fall prevention strategies.

## Introduction

Scales and instruments play an important role in health research and practice [1]. The Activities-specific Balance Confidence (ABC) Scale is used to measure the perception of balance confidence in older adults when performing daily activities [2].

The ABC was created at the University of Waterloo, Canada, in 1995 by Lynda E. Powell and Anita M. Myers with the objective of using a scale with more detailed and precise questions than the Falls Efficacy Scale [2,3]. The questionnaire has been cross-culturally adapted by the following countries: the United Kingdom [4], China [5], Germany [6], Turkey [7], Spain [8], Portugal [9], and Brazil [10]. Across these adaptations, several common patterns have emerged: (1) the scale consistently demonstrated strong psychometric properties, such as high internal consistency and test-retest reliability; (2) only minor cultural or linguistic changes were needed to enhance item clarity, while the original structure of the scale was preserved; and (3) the tool has shown good applicability in both community-dwelling older adults and clinical populations, particularly those with vestibular disorders. Additionally, the ABC Scale proved sensitive to changes following balance-related interventions, reinforcing its utility across diverse cultural contexts.

The ABC questionnaire consists of 16 items to be completed and can be self-administered or completed by an interviewer in person, by telephone, or online. It is commonly used and subjective, specifically designed to measure balance confidence and fear of falling that an individual perceives when performing specific activities such as walking around the house, going up and down stairs, getting in and out of a car, and bending to pick up an object from the floor, among others [2].

The Spanish version [8] was conducted in patients with vestibular disorders capable of understanding and completing the questionnaire. It demonstrated good internal consistency, test-retest reliability, construct validity, and concurrent validity. Additionally, it was able to discriminate between participants who reported one or more falls in the last 12 months and those who did not. In the cross-cultural adaptation process of the Spanish ABC, item 8 was modified to adapt to cultural differences. Among the 16 activities, items 6, 15, and 16 obtained the lowest confidence levels. These results agree with those described by other studies in different populations [4-10].

In the most recent validation and cultural adaptation of the ABC Scale, several modifications were implemented following recommendations from the original authors. One key reform involved simplifying the percentage choice list format from 11 possible responses to five (0%, 25%, 50%, 75%, or 100%), with a fixed 25% interval between options. This change, supported by three independent studies [11-13], was shown to improve probability curves and enhance usability, particularly for individuals with movement disorders, by adopting a checklist format (tick-based responses). Critically, the scoring method (summing percentages and dividing by 16) remained unchanged [11-13].

These psychometric refinements are especially relevant given the population the ABC Scale frequently targets: older adults. Falls represent a major public health concern in this group, with 30-50% of individuals aged 60+ experiencing at least one fall annually [2,14,15]. The consequences, ranging from functional decline (post-fall syndrome) to hospitalizations and mortality, underscore the need for reliable assessment tools like the adapted ABC Scale to evaluate balance confidence and guide interventions.

In Argentina, the most recent statistics (2012) highlight that at least 32% of adults over 65 years of age have experienced a fall and that, of this percentage, 56% have fallen more than once in the last two years. One of the most common consequences is the fracture of one or more bones, which frequently requires hospitalization. Of the total population that had at least one fall, 46% were between 60 and 74 years old, and 57% of people over 75 years old reported the fracture of at least one bone. These data suggest that a reduction in falls could decrease their consequences and the associated healthcare costs [16].

There are various factors that increase the risk of falls in older adults, one of the main ones being the lack of confidence to perform an activity, which, together with the fear of falling, leads to a restriction of activities of daily living, a decrease in physical conditioning and functional disability, which would further increase the risk of new falls [5].

The ABC questionnaire is used to measure the perception of balance confidence in older adults [10]. Based on the results obtained, individuals can be grouped by functionality level: low (less than 50%), moderate (between 50% and 80%), and high (greater than 80%). These cutoff points were originally proposed by Powell and Myers in the validation of the ABC Scale as a way to characterize different levels of balance confidence in older adults and are commonly referenced in the literature [2,17]. Although the Spanish version by Montilla-Ibáñez et al. [8] exists, it cannot be used in Argentina due to cultural differences found in the questions. While a cultural adaptation could be made, this version uses the format with 11 possible responses per question, which is currently not recommended by the original authors. Based on the above, the need for a culturally translated and adapted tool that assesses balance confidence in older adults in Argentina is evident in order to obtain an outcome variable in the implementation of prevention and treatment strategies to improve the quality of life of this population. The objective of this study was to translate, culturally adapt, and evaluate the internal consistency of the ABC Scale questionnaire for use in the Argentine adult population over 65 years of age.

## Materials and methods

This study was approved by the Bioethics Committee (approval 201 EUPeSe/18) and conducted within the framework of the Scientific Research, Development and Technology Transfer and Innovations Programme (CyTMA2) of Universidad Nacional de La Matanza.

Following recommendations from the original authors and current literature, we maintained the response scale with 25% increments (0%, 25%, 50%, 75%, and 100%) for each item. The Argentinian Spanish adaptation preserves the original 16-item structure of the ABC Scale. Consistent with the original version, the final score is calculated by summing the percentages of all responses and dividing the total by 16 [11-13].

Translation and cross-cultural adaptation process

**Figure 1:**
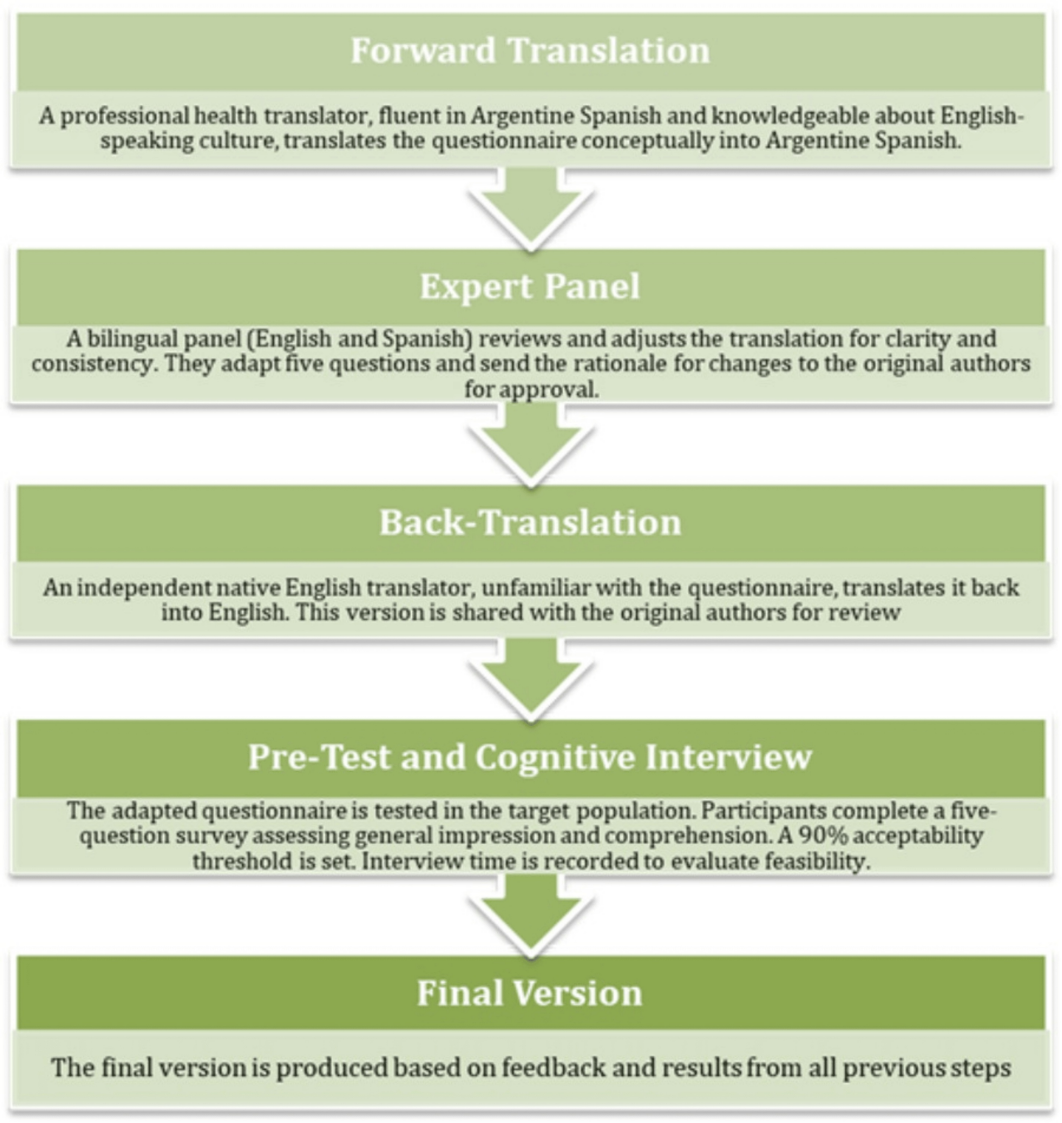
World Health Organization questionnaire translation process

The translation and cross-cultural adaptation process was performed according to the World Health Organization guidelines [18]. This process is detailed in Figure 1.

Forward Translation

A professional health translator familiar with the terminology in the area, knowledgeable about English-speaking culture, but with Argentine Spanish as their native language, translated the questionnaire conceptually into Argentine Spanish.

Expert Panel

A panel of bilingual experts (with knowledge of English and Spanish) was convened to identify and resolve inadequate expressions and concepts in the translation, as well as any discrepancies between the translation and existing or comparable versions of the questions. The product of this process was the adaptation of the questionnaire by modifying five questions from the original version. The original version authors received the rationale for the changes and approved them.

This panel was composed of five professionals: one otoneurologist, one neuro-otologist, one sociologist, and two vestibular therapists, all with over 15 years of experience in their respective fields. This multidisciplinary team ensured both the clinical and cultural relevance of the translation and adaptation process.

Back-Translation

Using the same approach described in the first step, the instrument was translated back into English by an independent translator who had no knowledge of the questionnaire and whose native language was English. This back-translation was also sent to the original version authors for their better understanding.

Pretest and Cognitive Interview

The instrument was pretested in the target population. For this purpose, an informed consent form was obtained, and a cognitive interview was conducted to assess participants’ understanding and acceptability of the scale [19]. The interview consisted of five structured questions applied to each of the 16 items in the questionnaire: (1) Was the question clear? If the answer was “no,” the interviewer followed up with, Why not? What was your difficulty? (2) Did you understand all the words in the question? (3) Could you express in your own words what the question is asking? (4) Do you think any changes are necessary to improve understanding? If so, which ones? (5) What was your overall impression of the question?

All responses were documented by the interviewer, and the interviews were recorded to allow review if needed. The results were then analyzed jointly by the interviewers and a panel of experts, who discussed participants’ responses and proposed modifications to improve clarity and cultural relevance. An acceptability threshold of ≥90% was established for satisfactory responses regarding general impression and comprehension [20]. Although not the main objective of this study, the administration time was also recorded to assess the tool’s feasibility in clinical practice, as the literature recommends a duration of less than 15 minutes [21].

Final Version

This was the result of the interaction of all the steps carried out and the pretest. As a result, the final version of the ABC translation for use in Argentina was obtained.

Participants

For proper cross-cultural adaptation, the literature recommends including at least 20 participants to assess questionnaire reliability [21,22]. Participants were recruited through convenience sampling among older adults who regularly took part in university extension activities. Informative sessions were held to present the project, and individuals who met the eligibility criteria were invited to participate voluntarily.

Inclusion criteria include native Argentine individuals aged over 65 years, of either sex, literate, and capable of walking independently with or without gait aids. Exclusion criteria include individuals who refused to provide informed consent; those with a history of vestibular or neurological disorders, whiplash injury, or lower limb pathology preventing assessment (e.g., diabetic polyneuropathy); individuals with communication or comprehension deficits scoring below 24 on the Mini-Mental State Examination (for those aged 65-75) or below 22 (for those over 75); and those unable to complete any of the study assessments for any reason.

All participants received verbal and written information about the study, and written informed consent was obtained prior to inclusion.

Statistical analysis

Continuous variables that assumed a normal distribution were reported as mean and SD. Otherwise, the median and IQR were used. Categorical variables were reported as frequencies and percentages. The Shapiro-Wilk test was used to determine the sample distribution. A p-value < 0.05 was considered significant. For data analysis, IBM SPSS Statistics for Mac OS, Version 24.0 (Released 2016; IBM Corp., Armonk, NY, USA) was used.

Ceiling and floor effects

Ceiling and floor effects were considered present if more than 15% of participants obtained the minimum or maximum value of the questionnaire [23].

Internal consistency

Consistency was evaluated using Cronbach’s alpha coefficient of the questionnaire scores. It was considered acceptable when the coefficient value was in the range of 0.7 and 0.95 [23].

## Results

Of the 16 items comprising the questionnaire, five underwent vocabulary changes to add terms more related to the country’s culture and socioeconomic reality, such as “supermarket” instead of “shopping” and “slippery pavement” instead of “snowy pavement”. Table 1 presents the items with their respective changes and justification.

**Table 1:** Modified items from the original Canadian version with the explanation for the change This table presents the original and adapted items from the ABC Scale as part of the cross-cultural adaptation process.

Original items	Modification	Explanation
3. Bends down and picks up a slipper from the front of the wardrobe floor	3. Bends down to pick up an object located on the floor	Due to cultural habits in Argentina, the word “slipper” was replaced with “object”. This minor change does not alter the original intent of bending down to pick up a familiar item.
10. Walks through a car park to the shopping centre	10. Walks through a car park to the entrance of a shopping centre, supermarket, or mall	To account for socioeconomic differences, the words “supermarket” and “mall” were added. This slight adjustment does not change the original intent of “walking through a car park...”.
12. Walks in a crowded shopping centre where people pass quickly by your side	12. Walks in a crowded place where people pass quickly close to them	Due to socioeconomic differences, “shopping centre” was replaced with “place”. This minimal change does not modify the original intent of “walking in a crowded area”.
13. People push you while walking through the shopping centre	13. Collides with or has physical contact with people	Again, due to socioeconomic differences, “shopping centre” was replaced with “busy place”. This minor change does not alter the original intent of “walking through a shopping centre”.
16. Walks outdoors on icy pavements	16. Walks on slippery pavements	Due to climatic and geographical differences in Argentina, the phrase “icy pavements” was replaced with “slippery pavements”. This does not change the original intent of “walking outdoors on icy pavements”.

The total analyzed sample consisted of 30 subjects, whose characteristics can be seen in Table 2. The time taken to administer the scale in the initial evaluation averaged two minutes and 17 seconds (SD: one minute).

**Table 2:** Characteristics of the study participants

Variables	N = 30
Female, n (%)	21 (70)
Male, n (%)	9 (30)
Age, median (IQR), years	69.5 (66-77.5)
Mini-Mental State Examination (IQR), points	28.21 (25-30)
Education level, n (%)	
Incomplete primary education	3 (10)
Complete primary education	20 (66.7)
Complete secondary education	7 (23.3)
Retired, n (%)	23 (76.7)
Not retired. n (%)	7 (23.3)

When evaluating questionnaire comprehension, it was observed that 100% of subjects responded satisfactorily. Regarding difficulty, 15 (50%) participants found it normal, and the rest rated it as easy or very easy. None of the participants reported the questionnaire as difficult or very difficult (Table 3).

**Table 3:** Summary of participants’ general impressions of the questionnaire

Variables	n = 30
Administration time, mean (SD), min:sec	02:17 (01:00)
Affirmative response, n (%)	
Did any questions seem unclear or confusing?	5 (16.7)
Did you have to imagine any activities?	13 (43.3)
Q6	4 (13.3)
Q13	4 (13.3)
Q14	2 (6.7)
Q15	6 (20)
Q16	3 (10)
Questionnaire difficulty level	
Very easy	8 (26.7)
Easy	7 (23.3)
Moderate	15 (50)
Difficult	0 (0)
Very difficult	0 (0)

Table 4 reports the frequencies for each question. The median ABC score was 83.6 (IQR 67.2-98.4) percentage points. A ceiling effect, defined as participants reaching the maximum score, was observed in five (16%) cases. The minimum value of 31.2 percentage points refers to the lowest total score obtained by a participant, expressed as a percentage of the maximum possible score. Internal consistency was acceptable (Cronbach’s alpha: 0.92).

**Table 4:** Item-specific and overall results from the ABC Scale questionnaire (n = 30) Values are expressed as the number of responses and percentage (%) except for the total score, which is presented as median (IQR). ABC, Activities-specific Balance Confidence

Question	0%	25%	50%	75%	100%
Q1	-	-	1 (3.3)	6 (20)	23 (76.7)
Q2	-	2 (6.7)	3 (10)	7 (23.3)	17 (56.7)
Q3	-	-	3 (10)	5 (16.7)	20 (66.7)
Q4	-	-	5 (16.7)	6 (20)	19 (63.3)
Q5	2 (6.7)	3 (10)	6 (20)	3 (10)	16 (53.3)
Q6	5 (16.7)	3 (10)	4 (13.3)	7 (23.3)	11 (36.7)
Q7	-	-	2 (6.7)	4 (13.3)	24 (80)
Q8	-	1 (3.3)	1 (3.3)	5 (16.7)	23 (76.7)
Q9	-	1 (3.3)	3 (10)	3 (10)	23 (76.7)
Q10	-	-	2 (6.7)	5 (16.7)	23 (76.7)
Q11	1 (3.3)	3 (10)	5 (16.7)	3 (10)	18 (60)
Q12	-	3 (10)	2 (6.7)	8 (26.7)	17 (56.7)
Q13	3 (10)	2 (6.7)	5 (16.7)	8 (26.7)	12 (40)
Q14	1 (3.3)	2 (6.7)	-	5 (16.7)	22 (73.3)
Q15	7 (23.3)	5 (16.7)	1 (3.3)	4 (13.3)	13 (43.3)
Q16	3 (10)	8 (26.7)	5 (16.7)	6 (20)	8 (26.7)
Total score					
Self-perceived balance confidence, median (IQR) %	83.6 (67.2-98.4)				

## Discussion

Five of the 16 items were modified for cross-cultural adaptation. The results indicate that most participants found the Argentine version easy to answer, taking approximately two minutes to complete the questionnaire.

Changes were necessary to adapt specific elements to the country’s geography and sociodemographic reality, which differ from the Canadian population [21,24,25]. For example, the original expression “shopping” was changed to “supermarket” or "shopping centre”; the expression “walking on snow” was changed to “walking on slippery pavements”, without changing the original ideas and aspects assessed by the questionnaire. These types of alterations from the original version are necessary in cross-cultural adaptation processes, which is why researchers from other languages and countries have also done so [5,8,10].

In the Argentine version, items 3, 10, 12, 13, and 16 were changed. The same items had to be changed in the Brazilian Portuguese version [10]; items 3 and 16 were also modified in the Chinese version [5]. In all cases, these adaptations must be understandable and applicable to the entire population, taking into account sociocultural characteristics [10].

Of the 30 participants, 70% were female, with a median age of 69.5 years. Similar results were found in previous studies, as the vast majority of them were conducted with older adult populations, who tend to be at higher risk of falls [5,8,10,26]. In fact, the ABC questionnaire also showed the ability to discriminate between older adults with high mobility versus those with low mobility. Other studies have confirmed the reliability and validity of the scale with older people [26,27].

The majority of participants in this study had completed primary education and were retired, characteristics consistent with previous studies [8,26]. Regarding the general impression of the questionnaire, 50% of participants considered its difficulty level to be moderate. The median perceived balance confidence was 83.6% (IQR: 67.2-98.4), indicating a moderate level of physical functioning [17]. This value was higher than those reported in Brazilian (81.7%) [10], Chinese (71.6%) [5], Spanish (63.52%) [8], and other studies with similarly aged populations [26]. One possible explanation is that our sample consisted of community-dwelling older adults engaged in university extension programs, who may represent a more active and health-conscious subgroup. Participation in social, educational, and physical activities has been associated with better functional performance, higher confidence levels, and lower fear of falling. Additionally, our exclusion criteria filtered out individuals with known balance-impairing conditions, which may have also contributed to the overall higher confidence scores.

These findings suggest that, despite a limited educational background, participants were able to comprehend and complete the PROM effectively. However, it is important to acknowledge that health literacy can influence the interpretation and response to PROMs. Studies have shown that individuals with lower health literacy may face challenges in understanding complex instructions or technical language, potentially affecting the validity of their responses [24].

Considering the highest response rate in the “100% confidence” option, item seven (sweeping the floor) obtained the highest confidence level, similar to what was observed in the Brazilian version [10], the Spanish version with vestibular patients, and another version with older people [24]. Similarly, items involving the action of walking freely or swaying from side to side (items 1, 8, 9, and 10) also obtained higher confidence levels. A similar result was observed in the Chinese version (items 1, 8, and 9) [5], the Brazilian version (items 1, 8, 9, and 10) [10], the Spanish version with vestibular patients (items 1 and 8) [8], and in the version with older people (items 1 and 8) [26,28].

Conversely, the lowest “100% confidence” rates were observed in items 16, 6, and 15. These findings are consistent with results from other studies in different languages and populations [8,10]. When asked whether they had to imagine any activity, thirteen participants (43.3%) responded affirmatively, as the task was not part of their usual routine. This is noteworthy since we opted to culturally adapt certain items without excluding them, whereas other authors have chosen to omit culturally irrelevant items entirely [8,26].

In terms of the total questionnaire score, a ceiling effect was observed in five participants (16.7%), while the minimum score was 31.2 percentage points, indicating no clear floor effect. Similar patterns - presence of ceiling effects and absence of floor effects - have been reported in other adaptations of the ABC Scale [8,24,25].

Regarding internal consistency analysis, in this study, it was acceptable (Cronbach’s alpha: 0.92) [25], representing excellent reliability according to Andresen’s [29] criteria for recommending disability outcome tools (≥0.80). Previous translations and validations [5,8] have shown values ranging from 0.91 to 0.98 (including the original Powell and Myers study [2]), which are consistent with our findings.

A potential weakness of this study was the 16% ceiling effect, which could be related to better physical conditioning of some participants, a factor that was not assessed in this study, such as their level of physical activity. In addition, the small sample size represents a limitation, as it restricts the generalizability of the findings. While our participants were community-dwelling and relatively independent older adults, the results may not be applicable to populations with specific health conditions or lower levels of functioning. Nonetheless, it is worth noting that self-reported parameters such as balance confidence may, in some cases, be more predictive of fall risk than more formal or complex measures [30].

The main strength is the provision of a new tool for assessing balance confidence, translated into Argentine Spanish, contributing to the clinical practice of healthcare professionals and mainly to the care of this population. Our findings demonstrate that the translation and adaptation process from the original version allowed participants to understand all items. Future research is needed to evaluate other psychometric properties, such as validation, and to observe its application in populations with diseases and other conditions that could compromise balance confidence.

## Conclusions

The original version of the ABC Scale was successfully translated and culturally adapted for use in the older adult population in Argentina. The Argentine version demonstrated moderate internal consistency and was easily understood and completed by participants, confirming its feasibility in clinical settings.

This culturally adapted tool provides healthcare professionals with a reliable instrument to assess balance confidence in older adults. Further studies are needed to validate the tool's effectiveness across diverse clinical populations, such as individuals with neurological disorders, musculoskeletal conditions, or cognitive impairments. Additionally, research should explore its integration into multifactorial fall prevention programs to assess its impact on reducing fall incidence among older adults.

## Appendices

**Table 5:** Activities-specific Balance Confidence Scale If you currently do not perform the activity in question, try to imagine how confident you would be if you had to do it. If you normally use a walking aid or similar support (or hold onto someone) to perform the activity, rate your confidence considering this support. If you have any doubts when answering any of these questions, please consult your therapist. How confident are you that you would not lose your balance or become unsteady when... For each of the following activities, please indicate your level of confidence when performing them by selecting the corresponding percentage from the scale below, ranging from 0% (no confidence) to 100% (complete confidence). Thank you very much for your responses.

Activity	0	0.25	0.5	0.75	1
1. Walking around the house?	☐	☐	☐	☐	☐
2. Going up or down stairs?	☐	☐	☐	☐	☐
3. Bending over to pick up an object from the floor?	☐	☐	☐	☐	☐
4. Reaching for a small object on a shelf at eye level?	☐	☐	☐	☐	☐
5. Standing on tiptoes to reach something above your head?	☐	☐	☐	☐	☐
6. Standing on a chair to reach for something?	☐	☐	☐	☐	☐
7. Sweeping the floor?	☐	☐	☐	☐	☐
8. Walking outside to a nearby parked car?	☐	☐	☐	☐	☐
9. Getting into or out of a car?	☐	☐	☐	☐	☐
10. Walking across a car park to the entrance of a shopping centre, supermarket, or mall?	☐	☐	☐	☐	☐
11. Going up or down a ramp?	☐	☐	☐	☐	☐
12. Walking in a crowded place where people are rushing past you?	☐	☐	☐	☐	☐
13. Being bumped into or jostled by people?	☐	☐	☐	☐	☐
14. Getting on or off an escalator while holding the handrail?	☐	☐	☐	☐	☐
15. Getting on or off an escalator while carrying packages (and unable to hold the handrail)?	☐	☐	☐	☐	☐
16. Walking on slippery pavements?	☐	☐	☐	☐	☐
